# Unbreaking Assemblies in Molecular Simulations with Periodic Boundaries

**DOI:** 10.1021/acs.jcim.2c01574

**Published:** 2023-05-12

**Authors:** Bart M. H. Bruininks, Tsjerk A. Wassenaar, Ilpo Vattulainen

**Affiliations:** † Department of Physics, University of Helsinki, P.O. Box 64, FI-00014 Helsinki, Finland; ‡ Groningen Biomolecular Sciences and Biotechnology Institute, University of Groningen, P.O. Box 72, 9700 AB Groningen, The Netherlands

## Abstract

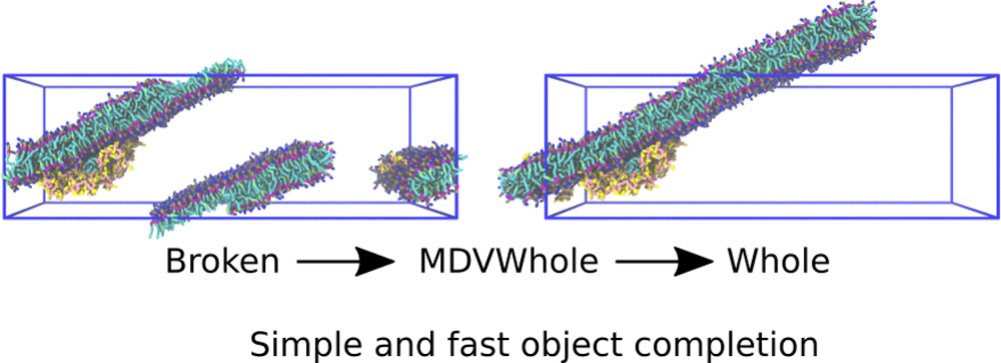

In molecular simulations, periodic boundary conditions are typically used to avoid surface effects occurring at the boundaries of the simulation box. A consequence of this is that molecules and assemblies may appear split over the boundaries. Broken molecular assemblies make it difficult to interpret, analyze, and visualize molecular simulation data. We present a general and fast algorithm that repairs molecular assemblies that are broken due to periodic boundary conditions. The open source method presented here, MDVWhole, works for all translation-only crystallographic periodic boundary conditions. The method consumes little memory and can fix the visualization of the assembly of millions of particles in a few seconds. Thus, it is suitable for processing both single simulation frames and long trajectories with millions of points.

## Introduction

Molecular dynamics (MD) simulations often involve a large number of molecules interacting with each other to form complex molecular assemblies.^1−14^ For instance, many proteins are functional in a multimeric state where they form an oligomeric nanoscale structure. On larger macroscopic scales one can observe complex lipid structures such as vesicles, lipid droplets, and mitochondrial membranes. Double-stranded DNA is an example of a molecular complex that spans all length scales, ranging from the nanoscale (width) to the macroscale (length).

MD simulation models are usually studied in the presence of periodic boundary conditions (PBCs). At the core of this technique is the simulated unit cell, which can be thought of as being regularly repeated throughout the space, so that the system to be simulated can be artificially modeled as having a finite size without physical boundaries. This method has been used successfully for decades, during which numerous methods have been developed to avoid/minimize possible artifacts. Thus, it is a bit surprising that although the first—and often the most illustrative—tool for the analysis of computationally studied molecular systems is visualization, there are still no methods for the visualization of simulations of complex systems that take PBCs into account in a satisfactory way.

**Figure 1:**
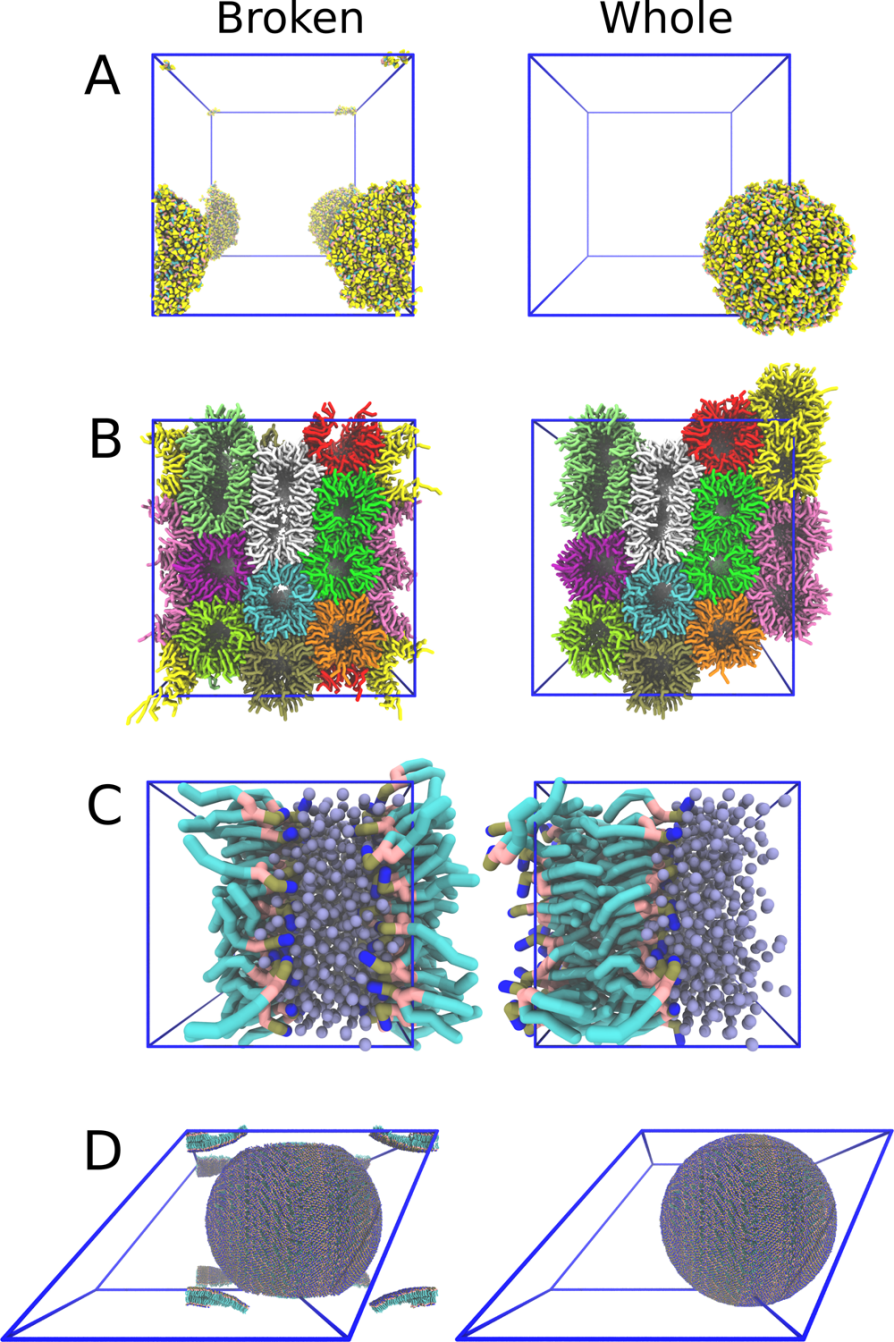
Object completion over periodic boundaries. The left-hand column (“Broken”) describes simulated systems using the standard representation where each molecule is completed and represented with at least one particle inside the simulation box, leading to the breaking of coherent molecular structures. The right-hand column (“Whole”) describes the same systems after the correction presented in this article has been made to their visualization. (A) An aggregate of dipeptides. (B) Lipids in the inverted hexagonal phase. Colors indicate different leaflets. (C) Lipid bilayer self-assembly. (D) A vesicle represented with dodecahedral periodic boundary conditions.

The core problem of using periodicity is that the simulated molecules and the structures they form appear fragmented at the boundaries of the unit cell. To fix this problem, solutions have been developed that work in certain conditions. For individual molecules, methods have been developed that allow the molecule to remain whole even when crossing the periodic boundary.^15−18^ Fixing broken molecules has the additional advantage of allowing the use of commonly available Euclidean geometric algorithms for the analysis of molecular properties. Moving on, the very recently developed FixBox^19^ algorithm completes and centers the object of interest in the simulation box. However, their method focuses on the presence of a single aggregate and is not tailored for handling trajectories. As FixBox relies on a single connected component’s segmentation, it also struggles with more complex assemblies such as the demonstrated inverted hexagonal phase (Figure 1 B). MDVWhole circumvents such problems by allowing the user to provide clustering information generated by any third party clustering algorithm. However, FixBox comes with some additional features when it comes to focusing the box around a single aggregate, which can be useful for visualization. This centering is currently not present in MDVWhole.

In this article, we present the open source tool MDVWhole and its underlying algorithm, with the help of which both individual molecules and the large assemblies they form can be visualized as a whole without breaking them due to PBCs. The used algorithm utilizes a voxel- and graph-based paradigm, which enables efficient processing in the general case for all periodic systems, extending to the representation of simulation trajectories formed by millions of particles and thousands of simulation frames (Table S1). It is obvious that this tool also facilitates the analysis of complex molecular systems.

Since the method is integrated with MDAnalysis^16,17^ and MDVoxelSegmentation,^20^ it can be used with most MD data types, and custom segmentation results can be used for object completion. The tool presented in this article is openly available at Github.

In the following sections, we first describe the general procedure to detect and complete molecular assemblies in the presence of PBCs. Then we show a number of case studies that illustrate the capabilities and performance of our method and its features. Finally, we discuss how our method can complement existing tools and workflows.

## The Algorithm

**Figure 2:**
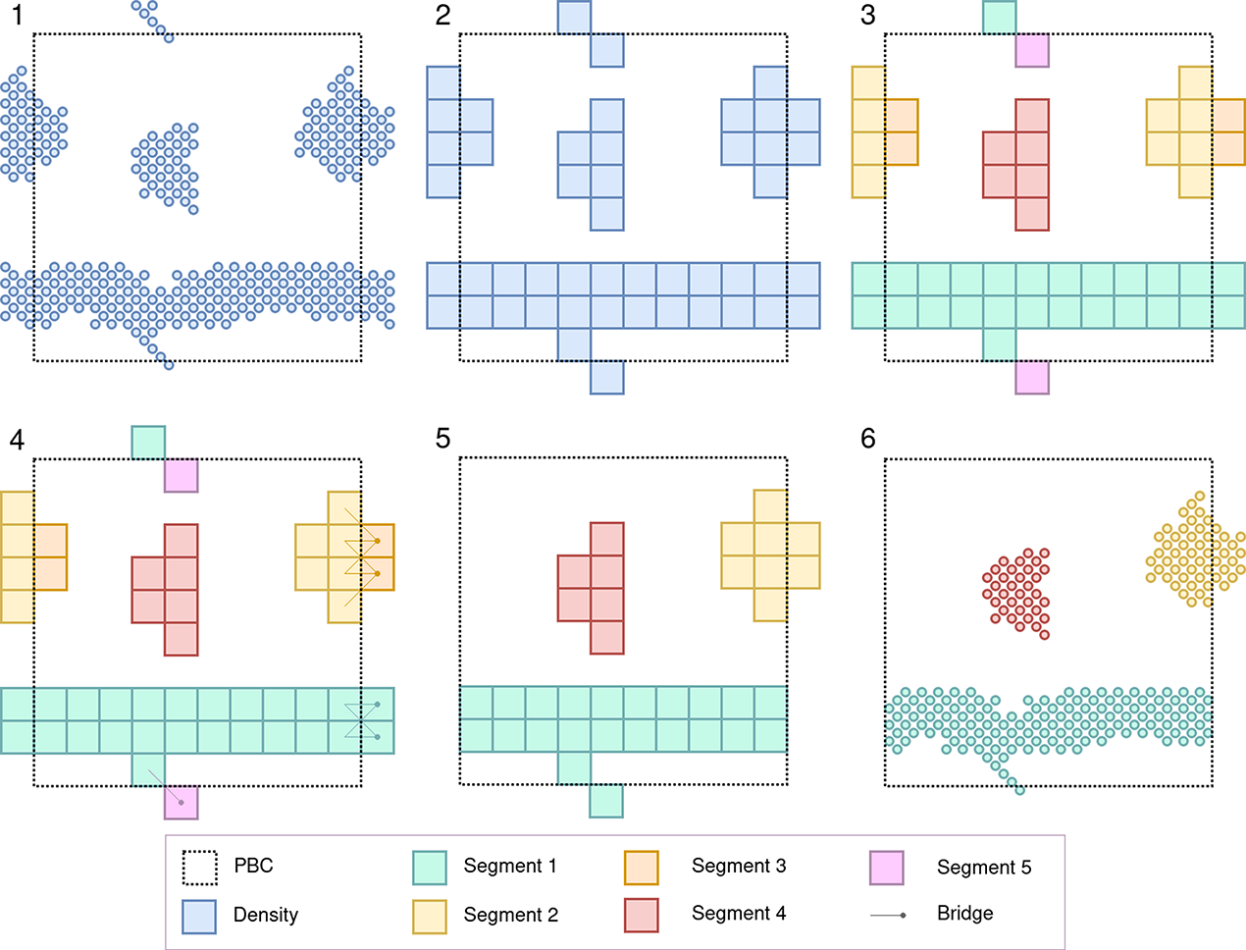
Schematic 2D representation of the MDVWhole algorithm. (1) The selected point cloud. (2) Conversion of point cloud into voxels. (3) Non-PBC segmentation of the voxels. (4) Connecting bridges are assigned between voxels at the box boundaries, using the periodic box definitions. (5) Segments are completed around their largest segment using the bridges. (6) The voxels are converted back to the point cloud.

The procedure of making point clouds whole over PBCs can be roughly divided into six steps (Figure 2).

1. First, the densities of interest are defined. This is done by using the -*selection* flag in the terminal command using the MDAnalysis selection syntax. By default this is “*not resname W WF ION*” (Figure 2.1). The default selection works well for most Martini^21,22^ systems as it selects everything except the most common solvent elements.
2. The selection of interest is voxelized using the previously proposed voxelization scheme.^20^ For voxelization, the resolution must be defined; it can be set using the -*resolution* flag (Figure 2.2). By default the resolution is 1; the units of the resolution depend on the units of the input file.
3. All voxel segments are defined (Figure 2.3). This is done based on density using the connected components segmentation (26 neighbors) or using a previously generated segmentation array. An example of such an array is the output of MDVoxelSegmentation.^20^ However, any *.npy file can be used, assuming the following dimensions [*n*_frames_, *n*_atoms_] with the segment IDs as their values. The custom array can be used by pointing to the path of the array using the -*clusters* flag. The use of a segmentation array will override the specified selection.
4. After segmentation, the periodic bridges between the segments (or within a segment) are detected using 26 neighbors per edge voxel. Only half of the edge voxels are evaluated, as all periodic contacts are symmetrical (Figure 2.4). A contact graph is generated using the defined segments as nodes and the PBC bridges as edges. Only the PBC shift between two segments with the highest occupancy is maintained. This trimming is important since it prevents rarely occurring (diagonal) bridges from being used in the subsequent path finding if a more dominant PBC connection is present (Figure S1). In the contact graph, segments connected by edges form the final objects, where multiple objects share no vertices or edges in the contact graph as they are disconnected.
5. The objects are completed around their largest segment (in voxels) by finding the shortest path from every segment to the largest segment they are connected to. The final shifts of the segments are defined as the sum of their PBC shifts defined by the bridges used in their shortest paths. This results in all objects that can be completed becoming whole (Figures 2.5 and 1A,D) and objects which are partially completable becoming as whole as possible (Figure 1B,C). For example, let there be an object broken into four segments forming the contact graph A–B–C–D, where A is the largest segment. Given that the PBC shift corresponding to bridge A → B is [+1*x*, 0*y*, −1*z*], the PBC shift corresponding to bridge B → C is [0*x*, −1*y*, 0*z*], and the PBC shift corresponding to bridge C → D is [−1*x*, 0*y*, 0*z*], from which it follows that the final PBC shift for D relative to A is [0*x*, −1*y*, −1*z*].
6. Finally the voxels are transformed back into a point cloud (Figure 2.6). It is important to note that since we are not using a nonperiodic displacement of the points, wrapping the points back into the periodic container will result in the original input. This reversibility guarantees that no information is lost during this analysis, as wrapping the output back into the box yields the input (except for machine-precision rounding errors).

The algorithm is described in pseudocode (Algorithm S1) and the full implementation for MDVWhole is available at Github and requires Python3.8 or later. The algorithm is performed on a per frame basis and the handling of molecular information is performed using MDAnalysis.^16,17^ Segmentation is implemented using SciPy.ndimage^23^ and for the graph logic, we use NetworkX.^24^ Numpy^25^ is used for handling arrays. MDVoxelSegmentation^20^ is used for voxelization and can be employed to generate more advanced segmentation.

## Results

To illustrate the use of MDVWhole in terms of case studies and its performance, we show examples of four different systems (Figure 1): aggregated proteins, a phase transition of dehydrated stacked bilayers to the inverted hexagonal phase, self-assembly of a lipid bilayer, and the formation of a vesicle. Each of these examples is described in more detail below. MDVWhole was used to complete all frames in the trajectories of these simulations, but here only a single representative frame is shown for each system. All presented data are available at the Zenodo database (10.5281/zenodo.7649132).

We will not describe in detail how the systems were created, since only the point cloud (trajectories) is/are relevant here. In this context, we only state that the simulation systems presented below are based on a coarse-grained (CG) Martini model.^11^ However, we emphasize that the method presented in this article can be applied to any particle-based simulation data. For completeness, we have uploaded the files used in these examples to an open access database, including the details of simulations, which are specified in the *.tpr files. We ran all tests on a Lenovo Thinkpad P1 (20TH-001AMX) with 32 GB of memory and an Intel Core i9-10885H (2.4 GHz) in Ubuntu 20. For timings we used a 500-frame trajectory and ran MDVWhole with the specified command; therefore, timings do include reading and writing of the frames/trajectories (Table 1).

**Table 1:** System Sizes and Performance

System	Total No. of Beads	Selected Beads	Timing (ms/frame)	Dimensions (*x*·*y*·*z*, nm^3^)
Dipeptides	174,149	34,500	168	26.3 · 26.3 · 26.3
Inverted Hexagonal	104,719	88,128	314	19.4 · 25.2 · 21.9
Vesicle	1,353,385	281,772	1,582	62.6 · 62.6 · 44.2
Self-Assembly	2,304	1,536	5	6.4 · 6.4 · 6.4
Self-Assembly (associative)	2,304	1,536	72	6.4 · 6.4 · 6.4

### Aggregate of Dipeptides

Polymer melts and ionic liquids are a hot topic these days because they can be used as a green alternative to non-environmentally friendly solvents.^26^ Here we show an example with a high quantity of dipeptides in solution forming a molecular aggregate (Figure 1 A). This system represents a “simple” single molecular aggregate, which is not periodically spanning any dimension. This system was also used to compare the speed of our algorithm to similar molecular completion algorithms. We found that aggregate completion by MDVWhole is slightly slower than molecular completion (Table S1).

#### Input Arguments

$ mdvwhole -f dipeptide_ball.gro -x dipeptide_ball.gro -o whole.gro

Using all of the default settings, the coordinate file and trajectory (-*f* -*x*) are successfully made whole for the solvated CG Martini systems.

### Phase Transition to a Complex Inverted Hexagonal Phase

The inverted hexagonal phase is a lipid phase which can be used to deliver drug molecules into cells. Here we show such an inverted phase made out of DOTAP and DOPE lipids (Figure 1 B). Results of this study have been published elsewhere.^10^ The inverted phase is a box-filling structure which spans the periodic boundaries in all directions.

#### Input Arguments

$ mdvseg -f inverted_hexagonal.gro -x inverted_hexagonal -eg none

$ mdvwhole -f inverted_hexagonal.tpr -x inverted_hexagonal.gro -clusters clusters.npy -o whole.gro

First the leaflet segmentation is performed using MDVoxelSegmentation^20^ (disabling any exclusions). Then the segments are made whole using the segments in the generated *clusters.npy*.

### Self-Assembly of a Lipid Bilayer

An old classic in the demonstration of the CG Martini force field is the self-assembly of a lipid bilayer in a water-like solvent (Figure 1 C). However, due to PBCs, the bilayer might appear fragmented when visualized. This visualization issue is one of the most often recurring topics at the cgmartini.nl forum, enforcing the importance of clear visualization.

#### Input Arguments

mdvwhole -f self_assembly.tpr -x self_assembly.gro -o whole.gro -sel “name C4A C4B D4A D4B C3A C3B D3A D3B″ -mol True -asso True -wa True -res −1

A file containing bonded information (i.e., *TPR*) is required for associative molecular completion. Due to the very small size of the system, we use only a subset of the lipid density for segmentation (e.g., lipid tails). This subselection is first made whole, after which all bonded components are associatively made whole as well (-*associative True*). Instead of associative completion, a more advanced segmentation than a single connected component could be used. For example, MDVoxelSegmentation^20^ uses multiple passes of masked connected components, where programs such as MemSurfer^27^ and FATSLIM^28^ use angle alignment on top of a distance criterion.

After object completion, some periodic segments might still have their molecules fragmented, as the segment cannot be made whole (i.e., a bilayer is often periodic in two dimensions). These molecules will be made whole as well (-*mol True*). All atoms of the input are written to the output, not only the selected ones (-*write_all True*). The -*resolution* is −1. Having a negative resolution allows for continuation if a certain frame cannot be voxelized with a voxel size deviation less than 5% of the target resolution, which is an issue in this system due to the small box size.

### Vesicle

A single solute in solution is best simulated under dodecahedral PBC conditions (the volume of a dodecahedron/cube with an equal-sized inner sphere is 0.707). However, this means that a vesicle with a diagonal equal to the shortest diagonal of the dodecahedron cannot be displayed whole in a triclinic box. However, since self-contacts are usually undesirable, a solvent layer having a minimum thickness of 4 nm surrounds the vesicle (Figure 1 D). Nevertheless, even with such a buffer zone the vesicle does not fit in the triclinic box.

#### Input Arguments

$ mdvwhole -f vesicle.gro -x vesicle.gro -o whole.gro

Default values were used for this system.

## Conclusions

We have shown that MDVWhole is a valuable tool for the postprocessing of data produced by molecular simulations. Object completion works for any point cloud in the space defined by PBCs, including atomistic and coarse-grained simulation models, as well as other particle-based ones. Due to the decoupling from the initial point cloud, the desired resolution can be adjusted for the analysis. This allows fast desktop-based PBC completion to be completed even on systems with millions of atoms/beads and thousands of frames. The memory requirements of the algorithm are modest because no additional copies of the simulation box are created during the analysis—that is a typical *ad hoc* approach to solve this issue. Even if such copying is not a bottleneck in postanalysis of small systems, it becomes problematic in large simulation systems and/or molecular assemblies that cross periodic boundaries more than once. In addition to object completion, the tool also supports molecular completion, making MDVWhole a general solution when dealing with PBC-related object fragmentation of single frames and trajectories.

Due to the close link between MDVWhole, MDAnalysis,^16,17^ and MDVoxelSegmentation^20^ as well as its availability on PyPi and Github, MDVWhole will be easy to pick up, even for less experienced users or users starting their research career.

## Data and Software Availability

The tool presented in this article is openly available at https://github.com/BartBruininks/mdvwhole. The data presented in this article are openly available at Zenodo: 10.5281/zenodo.7649132.

## Supporting Information Available

Performance (speed) comparison table for MDVWhole versus similar tools (Table S1), step by step view of completion of intrinsically periodic objects demonstrated with an undulated bilayer (Figure S1), and summary of the MDVWhole algorithm in pseudocode (Algorithm S1) (PDF)
