# Supplementary material for: Unbreaking Assemblies in Molecular Simulations with Periodic Boundaries
Source: J Chem Inf Model. 2023 May 12;63(11):3448–52. doi: 10.1021/acs.jcim.2c01574 (PMC10268952; doi:10.1021/acs.jcim.2c01574)
Supplement: Supplementary file 1 — ci2c01574_si_002.pdf [file ci2c01574_si_002.pdf]

Supporting Information - Unbreaking Assemblies in Molecular Simulations With Periodic Boundaries

Bart M. H. Bruininks,*,† Tsjerk A. Wassenaar,‡ and Ilpo Vattulainen*,†

†*Department of Physics, University of Helsinki, P.O. Box 64 FI-00014 Helsinki, Finland*

‡*Groningen Biomolecular Sciences and Biotechnology Institute, University of Groningen, PO Box 72 9700 AB Groningen, the Netherlands*

E-mail: bartbruininks@gmail.com; ilpo.vattulainen@helsinki.fi

Content

This document contains three entries. The performance (speed) comparison table for MDVWhole versus similar tools (Table S1). A step by step view of completion of intrinsically periodic objects demonstrated with an undulated bilayer (Fig. S1). Finally the MDVWhole algorithm summarized in pseudocode (Alg. S1).

The MDVWhole algorithm is openly available at:

https://github.com/BartBruininks/mdvwhole

The data presented in this manuscript is openly available at Zenodo:

https://doi.org/10.5281/zenodo.7649132

Supporting Information

Table S1: Performance comparison. The MDVWhole algorithm benchmarked against the molecular completions algorithms of GROMACS and MDAnalysis. We also compared against the object completion of Fixbox. The analysis was performed on the dipeptide system (500 frames) and repeated three times. The scripts used for generating benchmarks are included in the data set. However, keep in mind that the specified timings are system dependent and not all algorithm scale the same. For example FixBox does not include a trajectory option and therefore every frame in the trajectory was read from a GRO. For a single frame Fixbox can be faster than MDVWhole especially for small systems. The main point here is that the object completion can be performed at near real time for systems up to millions of beads, on a laptop/desktop.

Algorithm	Mean (s)	STD (s)
gmx mol whole	34.87	0.26
mda mol whole	79.27	0.73
fixbox	6066.93	0.56
mdvwhole	100.31	4.04

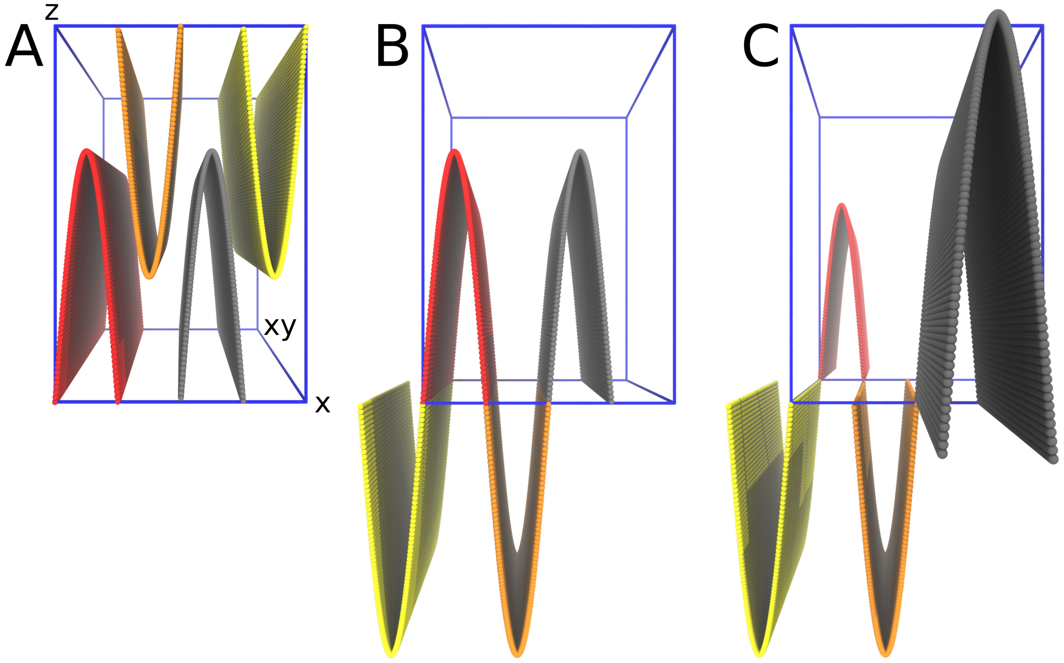

Figure S1: Completion of a heavily undulated membrane. A single object might be larger than a single dimension, without spanning it (A). Shown is a membrane which is spanning the xy dimensions (in plane with the membrane), and is larger than the z dimension in without spanning it. The membrane cannot be completed in the xy plane, but it can be completed in the z dimension. An object is considered whole if it is continuous, however, for objects which are intrinsically periodic this is ambiguous and multiple configurations are continuous (for example B and C). During the creation of the contact graph for the segments of an object we consider the dominant PBC connection between each pair of segments in an object. By doing so, we prevent low occupied diagonals to dominate the final result (C), even if a much larger (usually non-diagonal) connecting interface is present. In short in situations where the object cannot be completely made whole, we make it as whole as possible (B).

Algorithm S1 MDVWhole

```
Require: |atomgroup| > 0
  pbc ← atomgroup.box_dimensions
  ndimage ← discretize(atomgroup)
  segments ← label(ndimage)                                          ▷ Labeling is non-periodic
  edgevoxels ← []
  for edgevoxel in ndimage do
      edgevoxel.label ← labels[edgevoxel]
      edgevoxel.displacements,
      edgevoxel.labels ← pbc_neighbors(edgevoxel)                    ▷ 26 neighbors and periodic
      edgevoxels.append(edgevoxel)
  end for
  graph ← network.graph()
  graph.nodes ← unique(labels)
  graph.edges ← []
  for voxel in edgevoxels do
      for idx, nlabel in voxel.labels do
          edge1 ← [voxel.label, nlabel, direction = voxel.displacements[idx], weight = 1]
          edge2 ← [nlabel, voxel.label, direction = −voxel.displacements[idx], weight = 1]
          if edge1 not in graph.edges then
              graph.add_edges(edge1, edge2)
          else
              graph.edges[edge1, edge2].weight += 1
          end if
      end for
  end for
  sub_graphs ← graphs.sub_graphs()                                   ▷ Returns all fragmented assemblies
  for graph in sub_graphs do
      graph.edges ← trim_edges(graph.edges)                          ▷ Most prevalent edge |direction|
      central_segment ← biggest_label(graph.nodes)
      shortest_paths ← network.shortest_path(graph, central_segment)
      for path in shortest_paths do                                  ▷ The shortest path to a label
          displacements ← []
          for edge in path do
              displacement.append(edge.displacement)
          end for
          displacement ← sum(displacements)
          atomgroup[label].positions += pbc ∗ displacment
      end for
  end for
```
